# Low-dose human chorionic gonadotropin supplementation initiated at the onset of ovarian stimulation can improve oocyte quality without impairing endometrial receptivity: Case series

**DOI:** 10.1097/MD.0000000000032175

**Published:** 2022-12-02

**Authors:** Huizhen Lin, Xiaona Huang, Yue Zhao, Yangyang Wang, Shasha Wang, Fang Hong, Mei Pan, Liu Liu

**Affiliations:** a Center of Reproductive Medicine, Sir Run Run Shaw Hospital, School of Medicine, Zhejiang University, Hangzhou, PR China; b Key Laboratory of Reproductive Dysfunction Management of Zhejiang Province, Hangzhou, China.

**Keywords:** case report, hCG, ovarian stimulation, recurrent IVF failure

## Abstract

Whether continuous low-dose human chorionic gonadotropin (hCG) supplementation during controlled ovarian hyperstimulation (COH) can improve oocyte and embryo quality is still controversial in clinical practice.

**Patient concerns:**

We report the first case series of inadvertent COH in luteal-phase stimulation in the presence of endogenous or exogenous low-dose hCG.

**Diagnoses:**

Patients were diagnosed with infertility.

**Outcomes:**

The first two cases had inadvertent COH during preexisting pregnancy, and one of which produced more high-quality embryos (5 vs 1) in the presence of low hCG. Both cases had a live birth. The third case had 7 repeated failures of IVF, during which a total of 55 oocytes were obtained, but only 3 developed into transferable embryo. However, supplementation of 330 IU hCG per day from the onset of COH resulted in the recovery of one high-quality embryo and subsequent delivery of a healthy baby following fresh embryo transfer in eighth attemption.

**Lessons:**

In conclude, supplementation with low-dose hCG from the onset of ovarian stimulation can improve oocyte quality without impairing endometrial receptivity.

## 1. Introduction

It remains controversial whether the supplementation of low-dose human chorionic gonadotropin (hCG) improves oocyte quality. The clinical indication, timing of initiation and the optimal dosage of hCG for women undergoing in vitro fertilization (IVF) remains unclear. Relevant literature remains limited. A growing interest on the role of hCG on oocyte quality has derived from certain case reports of inadvertent stimulation in preexisting pregnancies. Some studies showed that elevated hCG compromised oocyte quality, possibly by causing premature luteinization.^[1–3]^ However, another study suggested that the presence of low hCG during controlled ovarian hyperstimulation (COH) increased the sensitivity of granulosa cells to gonadotropins, reducing the total dosage of gonadotropin required, resulting in more mature oocytes and high-quality embryos.^[4]^

One possible explanation for the different findings was that hCG levels differed in these studies. For example, in earlier reported cases (summarized in Table 1), hCG levels were already rather high [e.g., 1909 IU/L,^[1]^ 7867 IU/L^[3]^] at the commencement of COH. The higher levels of hCG may impair oocyte quality. Interestingly, another case studied by Goeckenjan et al^[4]^ reported that COH was initiated 8 days following fetal termination, and serum hCG was 300 IU/L at day 5 of stimulation. Therefore, hCG levels during the COH in this study was much lower than the 2 previous studies mentioned above.^[1,3]^ The patient reported by Goeckenjan et al was 37 years old and had a low anti-Mullerian hormone (AMH) level (1.6 ng/mL), yet 26 oocytes were obtained after COH. The authors concluded that the relatively low serum hCG would not lead to premature luteinization, and conversely, could promote androgen synthesis, up-regulate the expression of gonadotropin receptors, and enhance the sensitivity of granulosa cells to gonadotropins.

**Table 1:** Earlier reported cases which stimulates in preexisting pregnancy.

Author	Year	COH protocol	Years	AMH(ng/mL)	GP	MP	hCG(IU/L) (first mearsurement)	Days (when Gn was started)	Trriger	N. of oocytes(N)	N. of transferable embryos (N)	Outcome of preexisting pregnancy
Zech et al	2005	Long GnRH agonist	28	—	G1P0	Irregular	—	D27	hCG 10000 IU	3	1	Live birth
Goeckenjan et al	2013	GnRH antagonist	37	1.6	G1P0	-	300 (on D5 of stimulation)	8 d after abortion	Triptorelin 200 ug	26	—	—
Yavuz et al	2019	GnRH antagonist	28	—	—	Irregular	7867 (on D6 of stimulation)	D28	Leuprolide acetate 40 ug	3	3	Fetal termination
Castillo et al	2019	GnRH antagonist	29	—	G2P2	—	1904 (4 d after OPU)	D25	GnRH-α 0.2 mg	1	—	Fetal termination

AMH = anti-Müllerian hormone; COH = controlled ovarian hyperstimulation; GnRH = gonadotropin-releasing hormone, GP = gestation and parturition; hCG = human chorionic gonadotropin; MP = menstrual period, OPU = oocyte pick up.

As far as we know, there have been no published studies on inadvertent COH in luteal-phase stimulation during incidental pregnancy. In this paper, we report 3 cases, one with inadvertent COH in preexisting pregnancy that resulted in the recovery of more high-quality embryos (in the presence of low hCG) than in the first cycle (5 vs 1, respectively). Third case was characterized by repeated failure to recover viable embryos, with a total of 55 oocytes obtained, but only 3 that developed into transferable embryos in 7 previous attempts. In the eighth treatment cycle of the third case, daily supplementation of a low dose of 330 IU hCG from the onset of COH resulted in the recovery of one high-quality embryo, leading to the delivery of a healthy baby following fresh embryo transfer.

## 2. Case presentation

### 2.1. Case 1. High-quality embryos obtained in a cycle with a preexisting pregnancy

#### 2.1.1. Cycle 1. Progesterone-primed ovarian stimulation protocol.

**Figure 1.**
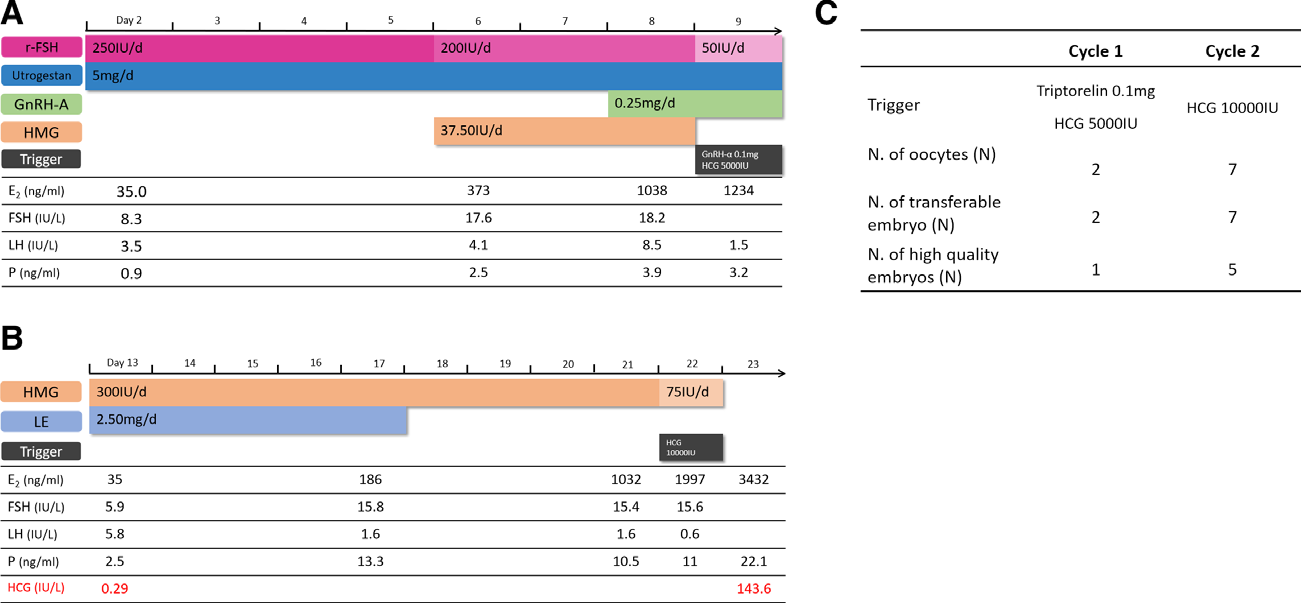
Details of the stimulation cycle of case 1. (A) Progesterone-primed ovarian stimulation protocol of cycle 1; (B) Inadvertent ovarian stimulation in a preexisting pregnancy of cycle 2; (C) Result of 2 cycles of case 1. Note: rFSH = recombinant follicle-stimulating hormone; GnRH-A = Gonadotropin-releasing hormone antagonist; HMG = human menopausal gonadotropin; GnRH-a = Gonadotropin-releasing hormone agonist; E2 = estradiol; FSH = Follicle stimulating hormone; LH = luteinizing hormone; P = Progesterone; LE = Letrozole; hCG = human chorionic gonadotropin.

The patient was 38 years old and had a body mass index (BMI) of 26.7. Her menstrual cycle phases were regular for 26–28 days and AMH level was 1.24 ng/mL. The patient’s right fallopian tube was obstructed, and her husband’s semen quality was normal. The couple came to our Reproductive Unit for IVF treatment. The first cycle was a progesterone-primed ovarian stimulation (PPOS) cycle (Fig. 1a). Daily injections of 250 IU recombinant follicle-stimulating hormone (rFSH, Puregon; Merck Sharp & Dohme, NJ) and 100 mg Utrogestan (Laboratoires Besins International, Paris, France) were given from the second day of the menstrual cycle. Then, 0.25 mg gonadotropin-releasing hormone (GnRH) antagonist (Orgalutran; Merck Sharp & Dohme, NJ) was given on day 8 of stimulation when luteinizing hormone (LH) levels had increased to 8.5 IU/L. Double trigger treatment with 0.1 mg GnRH agonist (Decapeptyl; Ferring, Malmo, Sweden) and 5000 IU hCG (Pregnyl; Organon, Oss, Netherlands) was given on day 9. In the first PPOS cycle, the patient produced 2 oocytes, resulting in 2 embryos, one of which was a high-quality embryo (Fig. 1c). However, the patient had no pregnancy after the subsequent frozen-thawed embryo transfer.

#### 2.1.2. Cycle 2. Inadvertent ovarian stimulation in a preexisting pregnancy.

The second cycle used a luteal-phase stimulation protocol (Fig. 1b). Daily injections of 300 IU human menopausal gonadotropin (HMG, Menotrophin for Injection; Renjian Pharmaceutical Group, Ningbo, China) and 2.5 mg (for the first 4 days) Letrozole (LE) (Hengrui Pharma, Jiangsu, China) were initiated from the 13^th^ day of the menstrual cycle. Progesterone levels fluctuated from 10.5 to 13.3 ng/mL during the mid-late follicular phase, and LH levels remained stable at approximately 0.6 to 1.6 IU/L. Then, 10,000 IU hCG was administered to trigger ovulation on day 9 of stimulation. The hCG level was 143 IU/L on the following day. The patient produced 7 oocytes, resulting in 7 embryos, 5 of which were high-quality embryos (Fig. 1c). The patient arrived at our hospital 17 days after oocyte retrieval as the patient had no menstrual cramps. A 1.5 × 1.3 cm gestational sac was detected by ultrasonography and the serum hCG level was 35558 IU/L. The patient was at 5 + 5/7 weeks of pregnancy. Therefore, implantation had occurred during COH in the presence of low hCG, which did not compromise the recruitment and growth of follicles, and may have improved oocyte quality. The preexisting pregnancy was not affected by COH and oocyte retrieval at an early stage. The patient delivered a healthy baby on January 5^th^, 2020.

### 2.2. Case 2. A serum hCG level of 17.1 IU/L on the trigger day

**Figure 2.**
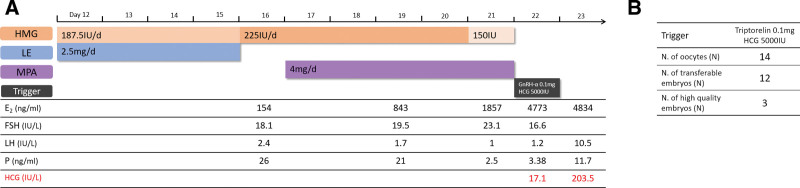
Details of the stimulation cycle of case 2. (A) Ovarian stimulation protocol; (B) the results of protocol. Note: MPA = medroxyprogesterone acetate.

The patient was 30 years old and had a BMI of 24.3 and had a spontaneous miscarriage at 8 weeks of pregnancy 2 years earlier. The patient’s menstrual cycle phases were regular for 40 days and the AMH level was 2.41 ng/mL. Her left fallopian tube was obstructed and the right one was patent. Her husband’s sperm quality was normal. The couple came to our Reproductive Unit for IVF treatment after one failed attempt of intrauterine sperm insemination. The couple had unprotected intercourse prior to the commencement of ovarian stimulation, and they did not wait until the next cycle because of the low chance of future conception. Daily injections of 187.5 IU HMG in combination with 2.5 mg LE (for the first 4 d) were given from the 12^th^ day of the menstrual cycle (Fig. 2a). The dose of HMG was adjusted according to follicular development. Medroxyprogesterone acetate tablets (4 mg/d; Shanghai Xinyi Balance Pharmaceutical, Shanghai, China) were given from day 6. Double trigger treatment with 0.1 mg GnRH agonist and 5000 IU hCG was administered on day 11 of stimulation. However, the serum hCG level was 17.1 IU/L on the trigger day. After being informed of the pregnancy and all possible related risks, the couple chose to continue the treatment. The patient produced 14 oocytes that resulted in 12 embryos, which included 3 high-quality embryos (Fig. 2b). Serum progesterone levels were high, around 21.0 to 26.0 ng/mL, and a significant drop was observed following 5 days of administration of medroxyprogesterone tablets. Serum LH levels remained stable and low, around 1.0 to 2.4 IU/L. Five days following oocyte retrieval, serum levels of hCG and progesterone were 644.6 IU/L and 53 ng/mL, respectively. Luteal support treatment was immediately started. The patient had additional hospitalization treatment for ovarian hyperstimulation syndrome for 1 week. The patient delivered a healthy baby on October 3^rd^, 2020.

### 2.3. Case 3 a healthy baby was delivered after low-dose hCG supplementation.

The patient was 33 years old, nulligravida and had a BMI of 22.0. The patient exhibited normal menstrual cycle phases for 26 days and the AMH level was 1.8 ng/mL. The couple came to our unit for IVF treatment following 3 failed attempts of intrauterine sperm insemination.

#### 2.3.1. Cycle 1. PPOS protocol.

**Figure 3.**
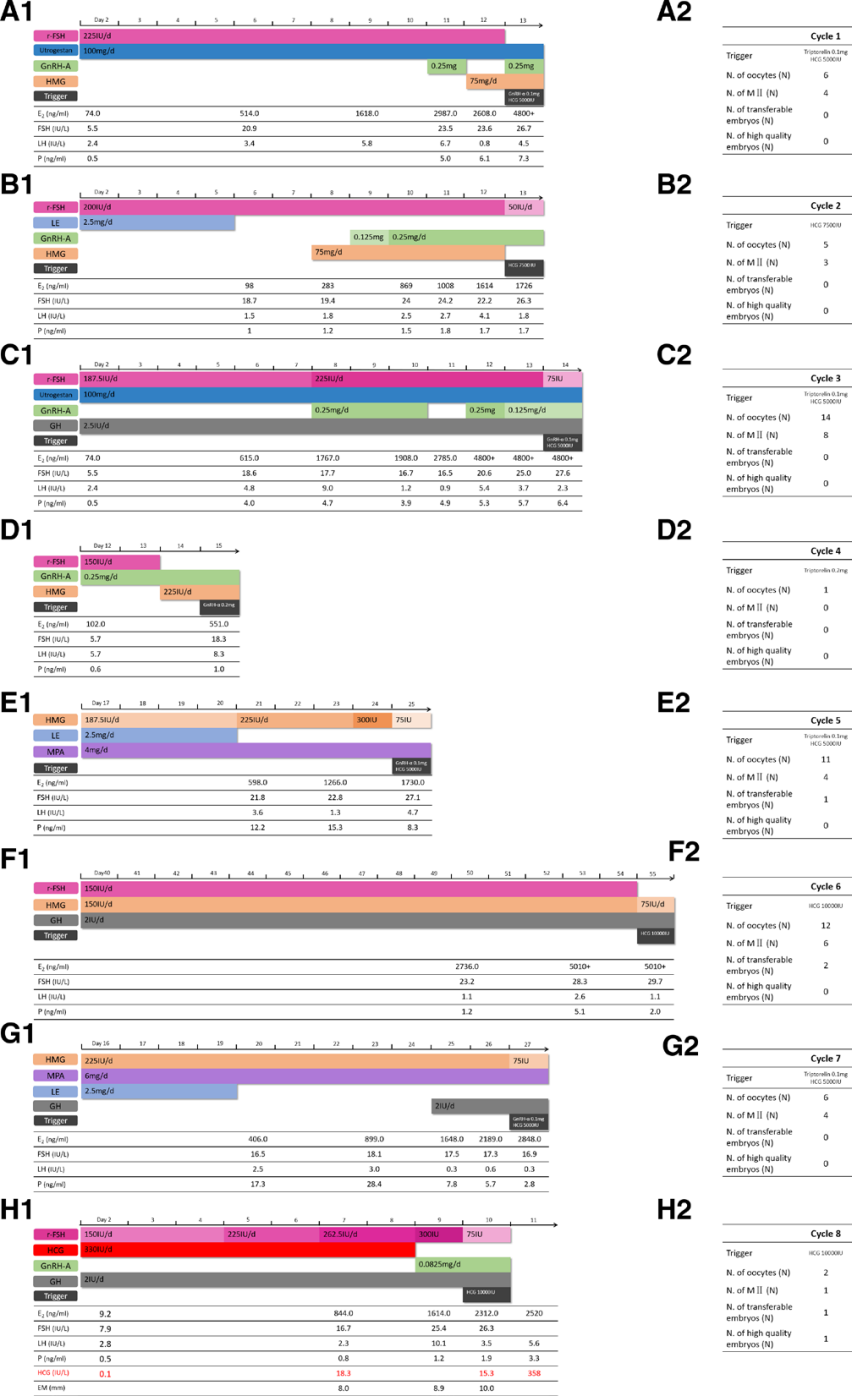
Details of the stimulation cycle of case 3. (a1)–(g1) ovarian stimulation protocols; (h1) Low-dose hCG supplementation in COH in a flexible GnRH antagonist protocol; (a2) – (h2) the results of protocols. GH = growth hormone; EM = endometrial thickness.

The first cycle was a PPOS protocol (Fig. 3a1). Daily injections of 225 IU Urofollitropin (Livzon Pharmaceutical, Guangdong, China) in combination with 100 mg Utrogestan were initiated from the second day of the menstrual cycle. Then, 0.25 mg GnRH antagonist was given on day 10 of stimulation when the LH level increased to 6.7 IU/L. A dramatic decrease in LH (to 0.8 IU/L) was observed immediately following the administration of GnRH antagonist. One day after stopping the use of antagonist, the LH level increased to 4.5 IU/L. Serum estradiol levels exhibited a similar changing pattern. Six oocytes were obtained, but none were fertilized following intracytoplasmic sperm injection (ICSI) (Fig. 3a2). Treatment with 100 mg/d utrogestan was not sufficient to inhibit the elevation of LH levels.

#### 2.3.2. Cycle 2. GnRH antagonist protocol.

The second cycle was a GnRH antagonist protocol (Fig. 3b1). Daily injections of 200 IU rFSH in combination with 0.25 mg LE (for the first 4 days) were initiated from the second day of the menstrual cycle. Then, 0.125 mg GnRH antagonist was given on day 8 of stimulation, then 0.25 mg/d GnRH antagonist was administered from day 9. Serum LH levels fluctuated from 1.5 to 4.1 IU/L. The increase of estradiol appeared to be slower than predicted, from 1008 to 1726 ng/L in the last 3 days. The patient produced 5 oocytes, 3 of which were mature metaphase II stage (MII) oocytes that were fertilized following ICSI, but none developed into transferable embryos (Fig. 3b2).

#### 2.3.3. Cycle 3. PPOS protocol.

Daily injections of 2.5 IU growth hormone (Jintropin; GenScis, Changchun, China) were administered for at least 1 month prior to the commencement of the third treatment cycle. The PPOS protocol was used again (Fig. 3c1). Then, daily injections of 187.5 IU Urofollitropin and 100 mg Utrogestan were given from the second day of the menstrual cycle. Again, 100 mg Utrogestan per day was not sufficient to inhibit the elevation of serum LH levels. GnRH antagonist (0.25 mg) was administered on day 7 when LH levels increased to 9.0 IU/L. An increase of serum estradiol was immediately inhibited following the administration of GnRH antagonist. Fourteen oocytes were obtained, 8 of which were mature MII oocytes, but only one was fertilized following ICSI, and it was not a transferable embryo (Fig. 3c2).

#### 2.3.4. Cycle 4. Modified natural cycle.

A natural cycle was performed to observe the quality of oocytes in the natural setting (Fig. 3d1). Follicle monitoring was performed from day 12 of the menstrual cycle, and daily injections of 150 IU Urofollitropin in combination with 0.25 mg GnRH antagonist were given from the same day. Then, 0.2 mg GnRH agonist was used to trigger ovulation. However, only one metaphase I stage oocyte was obtained (Fig. 3d1).

#### 2.3.5. Cycle 5. Luteal-phase ovarian stimulation cycle.

The fifth cycle was a luteal-phase stimulation cycle (Fig. 3e1). Daily administration of 187.5 IU HMG, 4 mg medroxyprogesterone, and 2.5 mg LE (for the initial 4 d) started from the 17^th^ day of the menstrual cycle. Serum LH levels fluctuated from 1.3–4.7 IU/L. Serum estradiol increased from 1266 pg/mL to 1730 pg/mL within the last 3 days of stimulation. In this cycle, eleven oocytes were obtained, 4 of which were mature MII oocytes. Three mature oocytes were fertilized following ICSI, but only one developed into a transferable embryo (Fig. 3e2).

#### 2.3.6. Cycle 6. GnRH agonist long protocol.

A long-acting GnRH agonist (3.75 mg Leuprorelin Acetate; Livzon Pharmaceutical, Shanghai, China) was administrated on the second day of the menstrual cycle. Daily injections of 150 IU Urofollitropin and 150 IU HMG were given 40 days later (Fig. 3f1). Then, 10000 IU hCG was administered to trigger ovulation. Serum LH levels remained stable at around 1.1–2.6 IU/L. Fresh embryo transfer was canceled because of the early elevation of progesterone levels prior to oocyte retrieval. Twelve oocytes were obtained, 6 of which were mature MII oocytes. Two transferable embryos (not high-quality) were available (Fig. 3f2) but failed to implant in the subsequent frozen-thawed embryo transfer cycle.

#### 2.3.7. Cycle 7. Luteal-phase ovarian stimulation cycle.

Daily administration of 225 IU HMG and 6 mg medroxyprogesterone, in combination with 2.5 mg LE (for the initial 4 d) was started from the 16^th^ day of the menstrual cycle (Fig. 3g1). Serum LH levels gradually decreased and the level was only 0.3 IU/L on the trigger day. The rising levels of estradiol peaked in the last 2 days of stimulation (from 2189 to 2848 pg/mL). Then, 0.1 mg GnRH agonist and 5000 IU hCG were administered for the final trigger on day 12. Six oocytes were obtained and 4 were mature MII oocytes, but none were fertilized following ICSI (Fig. 3g2).

#### 2.3.8. Cycle 8. Low-dose hCG supplementation in COH in a flexible GnRH antagonist protocol.

Daily administration of 150 IU Gonal-F (Merck Serono S.p.A., Rome, Italy) and 330 IU human chorionic gonadotrophin for injection (hCG; Renjian Pharmaceutical Group, Ningbo, China) was given from the second day of the menstrual cycle (Fig. 3h1). The dose of Gonal-F was adjusted according to the development of follicles. Then, 10000 IU hCG was given for the trigger on day 9 of stimulation. Consistent with an earlier study,^[5]^ serum hCG levels peaked at 18.3 IU/L following 6 days of daily administration. The LH level increased to 10.1 IU/L on day 8 of stimulation. After treatment with only 0.0825 mg GnRH antagonist, the LH dropped to 3.5 IU/L on the following day. Serum estradiol increased to suitable levels in the last 2 days of stimulation. Endometrial receptivity was not compromised by daily injections of 330 IU hCG. Endometrial thickness was 10 mm, type A, and the progesterone level was 1.9 ng/mL on the trigger day. In this cycle, the patient had obtained 2 oocytes, one of which was a mature MII oocyte that developed to a high-quality embryo (Fig. 3h2). The patient became pregnant following a fresh embryo transfer and delivered a healthy boy on September 14^th^, 2021.

## 3. Discussion

We reported 3 cases in this study. The first 2 cases underwent inadvertent COH during a preexisting pregnancy. We hypothesized that the low-level hCG in the COH process improved the quality of oocytes and embryos, and the ideal range of serum hCG was around 17 to 20 IU/L. By daily injection of 330 IU hCG to maintain the ideal range of serum hCG, the third case produced a high-quality embryo, and delivered a healthy boy following fresh embryo transfer.

The first case was 38 years old with an AMH level of 1.24 ng/mL. In the first cycle, serum LH levels fluctuated at relative high levels (Fig. 1a), but LH was at a rather low level in the second cycle. In this context, a low level of hCG in the second cycle provided the LH actions required to support the growth and development of oocytes, and further improved the quality of oocytes by unknown mechanisms. It is possible that hCG supplementation may produce better results than LH.

Indeed, a pilot randomized controlled study^[6]^ compared the live birth rates using daily injections of 150 IU urinary hCG or 150 IU rLH from day 6 of stimulation until the trigger day, in poor responders receiving GnRH antagonist protocols. The results showed that the live birth rate in the hCG group was 3.6 times higher than that in the rLH group. And in women with recurrent implantation failure, Drakakis et al^[7]^ found that women receiving supplementation of 200 IU hCG for the first 5 days of stimulation had a 3.6 times higher probability of achieving pregnancy compared with women receiving 200 IU rLH (95% confidence interval: 1.21–10.71, *P* = .022). A retrospective case-controlled study of women aged 40 years and older found that daily doses of 200 IU hCG for 4 days, starting from the first day of stimulation, resulted in a significantly increased number of retrieved mature and fertilized oocytes, as well as significantly higher estradiol levels on the day of hCG administration.^[8]^ Other studies examined the addition of hCG during the middle-late stage of ovarian stimulation. Decleer et al^[9]^ showed that 150 IU hCG per day from day 7 onwards, instead of highly purified HMG for ovarian stimulation, resulted in a significant reduction in the cost of IVF, and the clinical pregnancy rate was 38% higher in the hCG group than that in the HMG group. Madani et al^[10]^ conducted a prospective randomized pilot trial of poor ovarian responders, and used rFSH alone, 100 IU hCG and rFSH, or 200 IU hCG and rFSH, with hCG supplementation started from day 6 of stimulation. The number of MII oocytes retrieved was significantly higher in women with 100 IU hCG supplementation compared with the other hormone combinations.

According to some previous studies, hCG and LH are structurally similar molecules, but the beta subunits of hCG and LH have differing levels of post-translational glycosylation, which confers hCG a markedly increased half-life in comparison with LH (approximately 24–34 h vs approximately 30–60 min, respectively), a greater potency (6–8 times greater than that of LH) and more stable occupancy of the LH/choriogonadotropin receptor (LH/CGR).^[11]^ Besides, intracellular responses differ between LH and hCG stimulation of the LH/CGR.^[12–14]^ LH binding results in more potent activation of the proliferative and anti-apoptotic ERK 1/2 and AKT pathways, whereas hCG has a higher potency for activation of the steroidogenic cAMP pathways. Both LH and hCG fully promote testosterone production, whereas LH only partially stimulates progesterone production.^[14]^ In addition, LH and hCG lead to the activation of different genes in ovarian cumulus cells^[15]^ and altered cumulus cell expression profiles.^[16]^

Approximately 80% of small antral follicles with a diameter of 3 to 10 mm express LHR,^[17]^ and the expression level of LHR in granulosa cells can be stimulated by both FSH and LH/hCG. The LHR cDNA levels after ovarian stimulation were significantly higher among women receiving hCG compared with women receiving LH.^[7]^ It is interesting to note that the expression level of LHR can predict pregnancy and live birth outcomes in IVF.^[7,18]^ Because the LHR has a ubiquitous distribution in reproductive organs, hCG may stimulate endometrial angiogenesis and growth and extend the embryo implantation window, thus increasing pregnancy rates.^[19,20]^ In addition, hCG priming is thought to increase testosterone production, which in turn induces increased expression of FSH receptor in follicles, resulting in greater sensitivity to stimulation.^[8]^

A previous dose-response pilot study randomized normal responders into 4 hCG dose groups: 0, 50, 100, and 150 IU/d.^[21]^ Serum hCG levels reached a plateau following 6 days of daily administration, and hCG levels on the trigger day were 3.1, 5.5, and 11.0 IU/L in the 50, 100, and 150 IU/d groups, respectively. Estradiol concentrations reached maximum levels with 100 IU hCG/d. The highest number of top-quality embryos on day 3 per patient was from the group given 150 IU hCG/d. An association was found between the mean number of top-quality embryos per patient and serum hCG levels on day 6 of stimulation.^[21]^ As far as we know, there have been no previous studies that have used high doses of hCG like those administered in the current study. We found that daily injection of 330 IU hCG produced a serum plateau of approximately 17–20 IU/L without causing premature luteinization or impaired endometrial receptivity.

Huirne et al^[22]^ documented the importance of changing LH levels and showed that the magnitude of the reduction of LH during antagonist treatment was associated with low pregnancy rates, with no relevance to the actual LH concentrations. In the last treatment cycle of the third case in our study, only 0.0825 mg GnRH antagonist (1/3 the typical dose) was used when LH increased to 10 IU/L on day 8. The serum LH level on the following day was 3.5 IU/L and declined to a tolerable range. In the third case, 100 mg Utrogestan per day was not sufficient to inhibit the elevation of LH, whereas 6 mg medroxyprogesterone strongly inhibited LH. This finding highlights the important issue that individualized doses of progesterone in the PPOS protocol should be considered to better control the level of LH.

A recent randomized control study^[23]^ investigated whether the addition of low-dose hCG (100 IU/d) throughout stimulation of infertile women undergoing IVF improved IVF outcome parameters. Patients with a normal ovarian response were administered hCG supplementation from the onset of the follicular phase. The study found that low-dose hCG supplementation did not improve IVF outcome parameters. However, this result should be interpreted with caution because the study prematurely ended because of the Covid-19 pandemic. Further suitably powered and randomized controlled trials are needed in this area.

To conclude, low levels of hCG derived from a preexisting pregnancy improved oocyte quality, and individualized daily administration of 330 IU/L hCG resulted in a rather difficult case achieving pregnancy. Individualized doses of GnRH antagonist or progesterone should be considered. To fully address the association of serum hCG and pregnancy outcomes, it is necessary to conduct a robust randomized controlled trial using a higher daily dosage of hCG than those in previous studies, or a dosage determined by serum hCG in subjects with a poor prognosis for future fertility.

## Acknowledgments

We thank all patients enrolled in this case and Liwen Bianji (Edanz) (www.liwenbianji.cn/) for editing the English text of a draft of this manuscript.

## Author contributions

**Conceptualization:** Huizhen Lin, Liu Liu.

**Data curation:** Huizhen Lin, Xiaona Huang, Yue Zhao, Yangyang Wang, Shasha Wang, Fang Hong, Mei Pan.

**Writing—original draft:** Huizhen Lin, Xiaona Huang.

**Writing—review and editing:** Huizhen Lin, Liu Liu.

## Correction

The funding information has been changed from “The research was supported by the Project supported by the Natural Science Foundation of Zhejiang province, China (Grant No. HDMZ22H0410401), and the Public Projects of Zhejiang Province (Grant No. LGF20H80013)” to “This research was supported by the Huadong Medicine Joint Funds of the Zhejiang Provincial Natural Science Foundation of China under Grant No. LHDMZ22H040001, and the Public Projects of Zhejiang Province under Grant No. LGF20H80013.”
